# Validation of Treatment Escalation as a Definition of Atopic Eczema Flares

**DOI:** 10.1371/journal.pone.0124770

**Published:** 2015-04-21

**Authors:** Kim S. Thomas, Beth Stuart, Caroline J. O’Leary, Jochen Schmitt, Carle Paul, Hywel C. Williams, Sinead Langan

**Affiliations:** 1 Centre of Evidence Based Dermatology, University of Nottingham, Nottingham, United Kingdom; 2 Faculty of Medicine, University of Southampton, Southampton, United Kingdom; 3 MRC Clinical Trials Unit, London, United Kingdom; 4 Centre for Evidence-based Healthcare, Medical Faculty Carl Gustav Carus, TU Dresden, Germany; 5 INSERM 1056, Dermatology, Paul Sabatier University, Toulouse, France; 6 Faculty of Epidemiology and Population Health, London School of Hygiene and Tropical Medicine, London, United Kingdom; Shanghai Jiao Tong University School of Medicine, CHINA

## Abstract

**Background:**

Atopic eczema (AE) is a chronic disease with flares and remissions. Long-term control of AE flares has been identified as a core outcome domain for AE trials. However, it is unclear how flares should be defined and measured.

**Objective:**

To validate two concepts of AE flares based on daily reports of topical medication use: (i) escalation of treatment and (ii) days of topical anti-inflammatory medication use (topical corticosteroids and/or calcineurin inhibitors).

**Methods:**

Data from two published AE studies (studies A (n=336) and B (n=60)) were analysed separately. Validity and feasibility of flare definitions were assessed using daily global bother (scale 0 to 10) as the reference standard. Intra-class correlations were reported for continuous variables, and odds ratios and area under the receiver operator characteristic (ROC) curve for binary outcome measures.

**Results:**

Good agreement was found between both AE flare definitions and change in global bother: area under the ROC curve for treatment escalation of 0.70 and 0.73 in studies A and B respectively, and area under the ROC curve of 0.69 for topical anti-inflammatory medication use (Study A only). Significant positive relationships were found between validated severity scales (POEM, SASSAD, TIS) and the duration of AE flares occurring in the previous week – POEM and SASSAD rose by half a point for each unit increase in number of days in flare. Smaller increases were observed on the TIS scale. Completeness of daily diaries was 95% for Study A and 60% for Study B over 16 weeks).

**Conclusion:**

Both definitions were good proxy indicators of AE flares. We found no evidence that ‘escalation of treatment’ was a better measure of AE flares than ‘use of topical anti-inflammatory medications’. Capturing disease flares in AE trials through daily recording of medication use is feasible and appears to be a good indicator of long-term control.

**Trial registration:**

Current Controlled Trials ISRCTN71423189 (Study A).

## Introduction

Atopic eczema (AE) is a chronic relapsing skin condition that is characterised by periods of disease flare, followed by periods of relatively well-controlled disease [1]. In this regard it is similar to many chronic inflammatory conditions such as asthma or rheumatoid arthritis, where disease flare may be captured by escalation of treatment or symptoms [2–4]. For chronic conditions, assessment of disease control over time in clinical studies can be particularly challenging [5,6]. The concept of AE flares is one way of capturing disease chronicity, and may be a useful outcome for long-term, comparative effectiveness trials.

In recent years there has been growing interest in secondary prevention strategies for the management of AE, and prevention of flares has been advocated as a useful outcome measure in this context. The most extensive use of flare definitions in the AE literature is in relation to proactive treatment with topical corticosteroids or topical calcineurin inhibitors [7].

Two systematic reviews on how best to capture AE flares have shown that there is considerable variation in the definitions used to measure AE flares in clinical trials [5,8]. Many flare definitions rely on a physician’s assessment of the flare rather than assessment by patients, which are potentially more relevant but challenging to assess in long-term studies.

A review published in 2006 proposed a provisional definition of AE flares based on the need to escalate AE treatment in response to worsening of disease [8]. This definition assumed that escalation of treatment (or rescue therapy) was a good indicator of disease flares as it was a behavioural response to worsening of disease from the patient’s perspective. The proposed definition has now been used in several clinical studies; two of which have been used to inform this paper due to the availability of the study data[9,10] In this paper we describe and analyse our preliminary experiences of using both ‘*escalation of treatment’* and ‘*days of topical anti-inflammatory medication use’* as measures of AE flares. The results will be used to inform the Harmonising Outcome Measures for Eczema (HOME) initiative with regards to the most appropriate outcome measures to be used for the measurement of long-term control in clinical trials. The HOME initiative is an international collaboration working together to agree on a core set of outcome measures for use in all future AE clinical trials [11–13].

The specific aims of this study were: i) to assess the feasibility and validity of capturing AE flares from daily diary data in long-term studies; ii) to inform the “HOME Long-Term Control Working Group” in its consideration of the most appropriate way of capturing long-term disease control as part of a core outcome set for AE. Two primary hypotheses were tested: i) that days of *escalation of treatment* was a good indicator of overall disease control; ii) that *escalation of treatment* was a better indicator of long-term control than days of *topical anti-inflammatory medication use* (topical corticosteroids and/or topical calcineurin inhibitors).

## Methods

Ethics approval was not required for the study as analysis was based on existing datasets from previously conducted studies.

### Data available from original studies

Data from two UK-based studies including children with moderate to severe AE have been used to inform this analysis.).

#### Study A: Softened Water Eczema Trial (SWET) [10]

*A 4 month*, *observer blind*, randomised controlled trial involving 336 children with moderate to severe AE. Participants received normal care plus an ion-exchange water softener, or normal care alone. The primary outcome was AE severity, as assessed by blinded research nurses, using an objective severity scale [14]. Children, or their parent / guardian, completed paper diaries daily throughout the study (older children completed the diaries for themselves as appropriate).

#### Study B: Observational study to identify flare triggers [9]

A 6 month prospective cohort study involving 60 children with moderate to severe AE. The objective of the study was to assess the association between environmental exposures and disease flares in AE. Children or their parents completed daily electronic diaries capturing disease severity and environmental exposures. The primary outcome was a global “bother” score on a scale of 0–10 (where 0 represented no bother and 10 the most bother that they could imagine). This simple, patient-centred outcome was previously used in the development of a well validated AE severity scale [15]. In long-term studies of this kind, use of a simple scale minimises the daily burden on responders.

**Fig 1:**
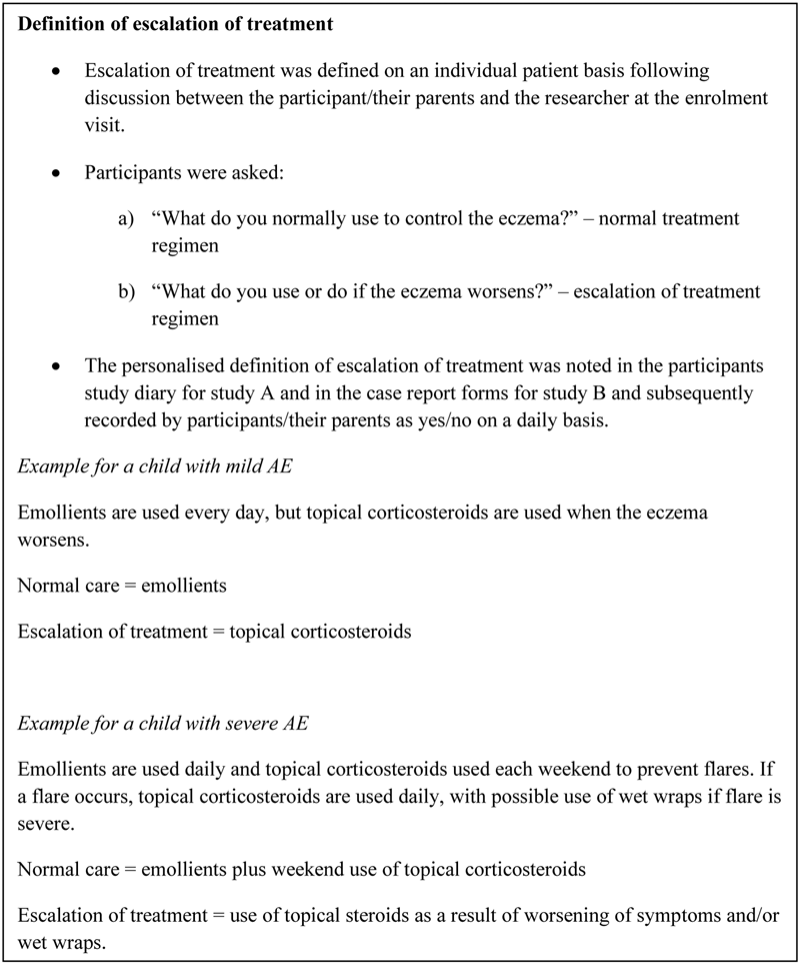
Definition of escalation of treatment.

For both studies, normal AE treatment and ‘escalation therapy' were defined on entry into the study, on an individual patient basis, following discussion with the patients and/or their parents or guardians (Fig 1). The decision to escalate treatment as a result of an exacerbation of AE was assessed daily as a binary yes / no response using either paper [10], or electronic [9] diaries. Electronic diaries allowed daily information to be entered up to midnight each day. Other outcomes that were collected on either a weekly or a monthly basis included the Patient Oriented Outcome Measure—POEM [15], Three Item Severity scale—TIS [16], and the Six Signs, Six Areas Atopic Dermatitis scale—SASSAD [14] in study A only.

### Key research questions

The HOME initiative has adopted the Outcome Measures in Rheumatology (OMERACT) filter[17] for the assessment of potential outcome measure for inclusion in the recommended core outcome set [12,18]. The OMERACT filter requires the assessment of three key features of potential outcome instruments: “truth, discrimination, and feasibility”.

The current study sought to evaluate our two definitions of AE flares (days when treatment was escalated and days of topical anti-inflammatory medication use), against the OMERACT filter (for truth and feasibility), in order to establish whether or not these outcomes could be useful in capturing long-term control of AE flares in future clinical trials. The following research questions were explored.

Truth: what is the flare definition measuring?
  1) What proportion of study days report an AE flare using the two flare definitions?
  2) How does the number of days in flare (as defined by the two flare definitions) relate to increases in self-reported AE ‘*global bother’*?
  3) How does the number of days in flare (as defined by the two flare definitions) correlate with other validated AE severity scales? [14–16]
  4) Is ‘*escalation of treatment’* a better indicator of *global bother* scores than ‘*topical anti-inflammatory medication*’?
Feasibility: is the scale feasible and acceptable for use in a variety of settings?
  5) How acceptable and easy to use is the concept of ‘escalation of treatment’ for participants and investigators?
  6) How complete are the two datasets, and did the use of paper diaries compared to electronic diaries influence the completeness of the data?

### Statistical methods

The sample size for this study was based on availability of data from the previous studies and no formal sample size estimation was conducted. Nevertheless, a sample size of >100 participants per analysis has been recommended as sufficient for validation studies [19].

The two datasets are reported descriptively, and have been analysed separately in order to explore the consistency of our findings across different datasets.

Two aspects of disease control were collected on a daily basis for 16 weeks in both studies: i) “How much bother has the AE caused today?”, and ii) “Has an escalation in treatment been required?” For Study A, use of topical anti-inflammatory medications (corticosteroids and/or topical calcineurin inhibitors) was also captured daily.

These daily data were used to calculate i) average ‘*global bother’* scores, ii) average number of days when treatment was escalated, and iii) average number of days of topical anti-inflammatory use. The average number of days in flare captured consecutive and non-consecutive days of flare in the same way. Thus an AE flare lasting a week was captured as seven days of flare in the same way as seven non-consecutive days of AE flare.

For analyses summarising the number of weeks in flare, a week was defined as being ‘in flare’ if a participant stepped up treatment, or applied topical anti-inflammatories, at least once in that week. When comparing the number of days in flare with existing validated scales, the number of days when treatment was escalated or anti-inflammatory medication was applied in the previous week was used.

Correlations between scales are reported using the intra-class correlation (ICC) for continuous outcome measures, and odds ratios (OR) with 95% CIs and area under the receiver operator characteristic curve (ROC) for binary outcome measures.

It was hypothesised that those with a higher *global bother* score on a given day would be more likely to *escalate treatment*, and that *escalation of treatment* would capture disease control better than *use of topical anti-inflammatory* medication.

In any week if a participant was missing data on whether or not they escalated treatment or used anti-inflammatory medication, we assumed that they did not do so. Missing values on any other key variables were treated as missing. Given the repeated measures nature of the study and the fact that many participants were missing at least one observation over the 16 week study period, the data were analysed using mixed models in Stata version 12.1 (e.g. xtlogit). This allows participants who have missing data to contribute information for any periods for which they have data.

Guidelines on essential features of studies to assess the psychometric properties of outcome scales have been followed in designing this study [19].

## Results

### Participants

Overall, 396 participants contributed to the analyses (n = 336 for Study A and n = 60 for Study B). Baseline characteristics of included participants are summarised (Table 1).

**Table 1:** Baseline characteristics of included participants.

	Study A	Study B
N enrolled	336	60
**Age, N (%)**		
Less than 3 years old	98 (29)	14 (23)
3 to 6 years old	123 (37)	13 (22)
7 years or older	115 (34)	33 (55)
Mean age (SD)	5.4 (4.1)	7.3 (4.8)
**Sex, N (%)**		
Male	193 (57)	32 (53)
Female	143 (43)	28 (47)
**Ethnicity, N (%)**		
White	260 (77)	38 (63)
Asian	33 (10)	14 (23)
Black	10 (3)	4 (7)
Mixed	19 (6)	4 (7)
Other	12 (4)	0
Not stated/unknown	2 (1)	0
**Eczema severity (POEM)** ^**1**^		
Mean (SD)	17 (6)	13 (7)
**Eczema severity (SASSAD), N (%)** ^**1**^		
10–19	143 (43)	N/A
>20	192 (57)	N/A
Mean (SD)	25.6 (13.6)	N/A
**Three Item Severity (TIS)**		
**Mean (SD)**	3.9 (1.8)	3.1 (1.5)
**Filaggrin status, N (%)**		
Presence of a mutation	94 (28)	10 (17)
Absence of a mutation	218 (65)	44 (73)
Unknown	24 (7)	6 (10)

^1^ There was 1 missing value for SASSAD in Study A, and I missing value for POEM in both Study A and Study B.

Abbreviations: Standard Deviation (SD), Patient Oriented Eczema Measure (POEM), Six Area, Six Sign Atopic Dermatitis (SASSAD), Not applicable (N/A)

### Truth: what are the flare definitions measuring?

*Research question 1*: What proportion of study days report an AE flare using the two flare definitions?

Summary data for all outcomes are summarised in Table 2.

**Table 2:** Summary of key outcome data over 16 week period of study.

			Study A	Study B
		n	336	59
**Bother score** (0–10)		Mean (SD)	4.45 (2.35)	3.46 (2.70)
		Median (IQR)	4.48 (3.26, 5.83)	3 (1, 5)
**Days in flare**	**Escalation of treatment**(0–112 days)	Mean (SD)	23.26 (25.06)	22.64 (19.54)
		Median (IQR)	15 (3,37)	18 (8.50, 30.50)
	**Anti-inflammatory medication used**(0–112 days)	Mean (SD)	49.60 (36.00)	N/A
		Median (IQR)	43.50 (19,73.75)	N/A
**POEM** (0–28 points)		Mean (SD)	12.52 (6.70)	10.08 (6.31)
**TIS** (0–9 points)		Mean (SD)	2.67 (1.82)	2.38 (1.68)
**SASSAD** (0–108 points)		Mean (SD)	20.31 (13.14)	N/A

Abbreviations: Patient Oriented Eczema Measure (POEM), Six area, six sign atopic dermatitis (SASSAD), Not applicable (N/A), Three item severity score (TIS).

**Fig 2:**
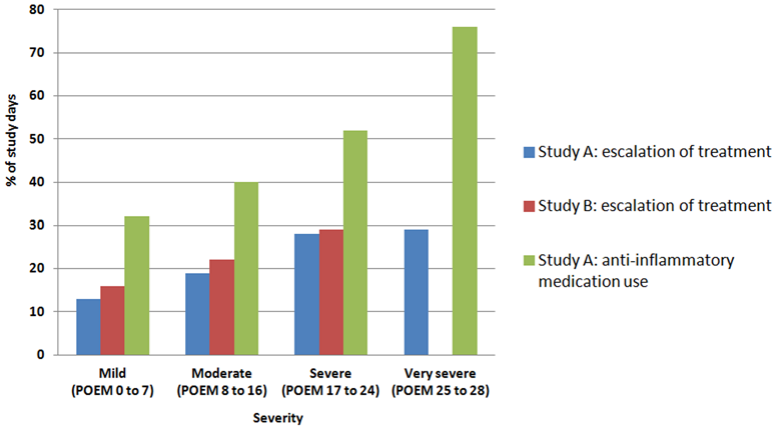
Proportion of days in flare by mean POEM scores for AE severity.

Throughout the 16 week study period, participants reported a flare based on *escalation of treatment* on 21% of the study days (23/112 days) for both Study A and Study B. This compares with 44% of days (49/112 days) when *anti-inflammatory medications* were used (data from Study A only). The proportion of days in flare by disease severity are summarised (Fig 2).

Research question 2: How does the number of days in flare relate to increases in self-reported ‘global bother’?

Overall there was a fairly good level of agreement between the *global bother* score and the measure of flare based on *escalation of treatment*, with an area under the ROC curve (sometimes called the “c-statistic”) of 0.70 (95% CI 0.69, 0.71) for Study A and 0.73 (95% CI 0.71, 0.74) for Study B. There was a similar level of agreement between the *global bother* score and days of *topical anti-inflammatory medication use* in study A, with a c-statistic of 0.69 (95% CI 0.67, 0.69).

To assess the impact of incremental changes in the *global bother* score, we compared the odds of *escalating treatment* and the odds of using *topical anti-inflammatory medication* by daily bother score (Table 3). The mean overall *global bother* score for Study A was 4.45 (SD 2.35), whilst for Study B the mean was 3.46 (SD 2.70), so a *global bother* score of four was chosen as the baseline for comparison. In both datasets, participants with *global bother* scores of less than four were significantly less likely to *escalate treatment* than those with the average score for each study of 4 (p<0.0001 for all comparisons), whilst those with a *global bother* score of more than four were significantly more likely to escalate treatment than those with the average score. Similar results were found for topical anti-inflammatory medication use (p<0.0001 for all comparisons) (Table 3).

**Table 3:** Odds of experiencing an AE flare (escalation of treatment) and odds of using anti-inflammatory medication by self-reported bother scores each day.

	Escalation of treatment	Use of anti-inflammatory medication*	
**Bother score**	**Study A (n = 334)** Odds ratio (95% CI)	**Study B (n = 59)** Odds ratio (95% CI)	**Study A (n = 334)** Odds ratio (95% CI)
0	0.01 (0.004, 0.01)	0.08 (0.06, 0.11)	0.05 (0.04, 0.07)
1	0.04 (0.03, 0.05)	0.15 (0.11, 0.21)	0.07 (0.05, 0.08)
2	0.19 (0.16, 0.23)	0.27 (0.21, 0.35)	0.25 (0.22, 0.29)
3	0.42 (0.37, 0.49)	0.63 (0.50, 0.80)	0.51 (0.46, 0.57)
4	1.00	1.00	1.00
5	2.16 (1.90, 2.45)	1.43 (1.11, 1.84)	1.84 (1.66, 2.05)
6	4.06 (3.55, 4.65)	2.73 (2.05, 3.65)	3.23 (2.87, 3.63)
7	7.78 (6.70, 9.03)	4.21 (3.08, 5.76)	5.46 (4.77, 6.24)
8	13.24 (11.21, 15.64)	6.43 (4.43, 9.35)	6.10 (5.22, 7.12)
9	19.36 (15.67, 23.92)	6.91 (4.41, 10.81)	6.23 (5.08, 7.64)
10	34.18 (25.54, 45.73)	7.34 (4.69, 11.49)	8.47 (6.37, 11.27)

*Topical corticosteroid / topical calcineurin inhibitors

When data are examined according to a change in bother score relative to the previous day, participants were twice as likely to *escalate treatment* or to *use topical anti-inflammatory medication* if they experienced a one point worsening of the *global bother* score, or four times more likely to *escalate treatment* or to *use topical anti-inflammatory medication* if the *global bother* score worsened by two points or more compared to those who did not have any change in their bother score or who improved. All results were statistically significant at p<0.0001 (Table 4).

**Table 4:** Odds of escalation of treatment and odds of using topical anti-inflammatory medication by change in bother score compared to the previous day.

	Escalation of treatment		Use of anti-inflammatory medication
**Change in Bother score**	**Study A (n = 334)** Odds ratio (95% CI)	**Study B (n = 59)** Odds ratio (95% CI)	**Study A (n = 334)** Odds ratio (95% CI)
No change or improved	1.00	1.00	1.00
1	2.01 (1.85, 2.18)	1.87(1.45, 2.41)	2.42 (2.25, 2.60)
2 or more	3.92 (3.47, 4.43)	3.17 (2.50, 4.03)	4.25 (3.81, 4.75)

Research question 3: How does the number of days in flare correlate with other validated AE severity scales?

There was a significant relationship between increasing severity as demonstrated using validated AE severity scales and the number of days when *escalation of treatment* was required in the preceding week. For POEM and SASSAD AE severity scales, both outcomes rose by about half a point for each unit increase in the number of days with a flare. For TIS, a small positive relationship was observed, but this was not significant (Table 5). The relationship between the AE severity scales and the use of *topical anti-inflammatory medication* was similar with POEM and SASSAD, increasing by about half a point for each 1-day increase in the number of days on which topical anti-inflammatory medication was used in a week. There was also a small, statistically significant increases in the TIS (Table 5).

**Table 5:** Mean increase in AE severity scales per unit increase in number of days when treatment escalated and use of a topical anti-inflammatory.

	Study A *Escalation of treatment (95% CI)	Correlation coefficient	Study B * Escalation of treatment (95% CI)	Correlation coefficient	Study A *Anti-inflammatory medication (95% CI)	Correlation coefficient
n	331		59		331	
POEM	0.51 (0.33, 0.69); p<0.001	0.527	0.63 (0.10, 1.16); p = 0.021	0.609	0.55 (0.40, 0.71; p<0.001)	0.528
TIS	0.04 (-0.01,0.09); p = 0.138	0.551	0.08 (-0.07, 0.22); p = 0.321	0.61	0.06 (0.02, 0.11; p = 0.003)	0.545
SASSAD	0.43 (0.14, 0.71); p = 0.004	0.762	N/A	N/A	0.53 (0.28, 0.78; p<0.0001)	0.757

*Increase in outcome measure for one unit increase in number of days in the previous week that treatment was escalated of topical anti-inflammatory medication was used. Uses data from weeks 4, 12 and 16

*Research question 4*: *Is* ‘*escalation of treatment’* a better indicator of *global bother* scores than ‘*topical anti-inflammatory medication*’?

It is likely that a flare definition based on use of anti-inflammatory medication may have overestimated the number of AE flares (44% of study days were classed as flare), and that this definition is potentially more likely to be influenced by variations in adherence to topical medications. Nevertheless, contrary to our original hypothesis, days of *topical anti-inflammatory medication use* performed just as well as days when *treatment was escalated* for all analyses, hence could be considered as an alternative to the *escalation of treatment* definition of AE flares.

### Feasibility: is the scale acceptable for use in a variety of settings?

Informal feedback from study participants was that completing the diaries on a daily basis was acceptable and relevant to the management of their AE. In some cases participants valued the use of a tool that allowed AE severity to be tracked over time. Since the need to escalate treatment in response to an AE flare was individualised to each participant, this was discussed face-to-face with participants (or their parent /guardian) on entry into each study. The types of treatment used as interventions to control AE flares varied, from use of mild topical corticosteroids through to the use of wet wrap dressings. For participants using systemic therapy, the need for additional topical treatments in addition to the systemic therapy was used to define worsening of the eczema, as systemic therapy is unlikely to be changed on a daily basis in response to changing disease activity.

Potential problems in using daily diaries to collect long-term disease control data included: the burden of data collection for participants (and resulting data management for the research team); potential confusion for participants about what is classified as “treatment escalation” if their treatment regimen changes during the period of the study; potential confusion over dates of data entry; and the restrictive data entry window for the electronic diaries that prevented data capture the following day.

The amount of missing data varied between the two studies. Data collection was more complete for Study A than Study B. For Study A, 95% of all possible daily data points were completed at 3-months and 94% of the possible daily data points were completed at 4-months, whereas for Study B, the number of complete data points at 3-months was 63% and at 4-months was 60%. The lower completion rates for Study B likely reflects the use of electronic diaries that prohibited completion after midnight each night.

## Discussion

### Main findings

The flare definition based on the need for escalation of therapy, first proposed by our group in 2006[8], has been explored in two clinical studies. Lessons have been learned in relation to the validity and feasibility of *escalation of therapy* as an indicator of flares in an applied setting. This study also provided an opportunity to evaluate a second measure of long-term control, namely daily *use of topical anti-inflammatory medication*.

In general our behavioural definition of AE flares, based on escalation of AE treatment, performed relatively well. The scale had face validity, in that the need to escalate treatment was intuitively understood by participants and investigators, and good construct validity, as the proportion of days in flare correlated positively with changes in ‘bother scores’ and validated severity scales (SASSAD and POEM). The difficulty of interpreting traditional AE severity scores has been highlighted by others [20], and having an outcome that is based on the number of days a patient has experienced an AE flare is a concept that is easily understood by both clinicians and patients.

### Escalation of therapy or use of topical anti-inflammatory medication as a measure of AE flares?

Contrary to our original hypothesis that *escalation of therapy* was a better measure of disease control than *use of topical anti-inflammatory medications*, the current study found that both scales performed well (although data on daily anti-inflammatory medication use was only available in one of the two datasets). If replicated in other settings, this finding is important, as it is potentially easier to collect the number of days when anti-inflammatory medication has been used, than it is to collect the number of days when therapy was escalated (as the latter requires individualised assessment for each participant, and may change during the course of a long-term study). Simply capturing days of anti-inflammatory medication use will also capture those using proactive treatment for the prevention (rather than treatment) of flares, and it is possible that such practice was less prevalent during the period when these two studies took place.

Some issues also remain with regards to the reliability and repeatability of the self-reported *escalation of treatment*, as it is not possible to establish whether participants in these studies interpreted the need to escalate treatment consistently throughout the study period. It is also possible that using an individualised approach to flare definition could make it more difficult to compare different studies and to pool data in meta-analyses. In this regard, asking participants to record whether or not they have applied topical anti-inflammatory medication on a daily (or weekly) basis, can also be challenging. Participants who are responding well to a given intervention may reduce the potency of their topical medication without reducing the total amount used; making it difficult to interpret data based solely on days of use. Variation in practice may also mean that some patients will have been advised to treat each flare episode aggressively, whilst others may not, with fear of side effects from topical corticosteroids affecting adherence to treatment independent of disease activity [21,22]. As an alternative to daily data collection of medication use, some AE trials have measured the total amount of topical medication used, either by weighing returned tubes or by recording the number of prescriptions issued. Further work would be helpful to establish the validity and feasibility of this approach, and to evaluate the accuracy of self-reported medication-use data through comparison with electronic monitors [23].

Although the included studies were of 4- and 6-months’ duration, the amount of missing data reported was surprisingly low, and patients appeared willing to complete the daily dairies for relatively lengthy periods of time. Nevertheless, the discrepancy between the number of missing data items in paper diaries compared to electronic diaries would suggest that some data may have been entered retrospectively in the paper diaries. This could be acceptable if the entries were completed within a couple of days of the flare, and it is likely that the electronic diaries were unduly strict in defining the time window for each day. With the increasing availability of, Smartphone technologies and, it is likely that a future application could be developed to address these issues.

### Relevance to other literature

Others have adopted similar behavioural definition of AE flares, based on the need to escalate treatment as a result of worsening disease [24–29], although the exact wording of these definitions has not been formally validated.

For other allergic diseases there is growing awareness of the need for core outcome sets. In the field of asthma, disease exacerbations have been identified as a core domain for inclusion in all future asthma trials [2]. It has been proposed that these should be captured using behavioural markers of exacerbation, such as use of systemic steroids, hospital admissions, emergency department visits and death [2]. Whilst AE flares are rarely of sufficient severity to warrant hospital admission or emergency care, this definition of an exacerbation shares the same concept of being a behavioural response to a worsening of the disease. For some AE studies, escalation of treatment could reasonably be interpreted as a need to seek health care advice, but in most cases accessing of health services is unlikely to be sensitive to change, and is probably not ideal as a single measure of flare.

### Strengths and weaknesses of this study

Strengths of this study are that daily data on escalation of therapy and topical medication use were available from two independent studies. The two datasets were examined and reported separately, and the associations identified in Study A were replicated in Study B. However, as the data from these studies was originally collected for another purpose, our analyses were determined by data availability. For this reason, the suggestion that topical medication use on a daily basis may be a useful indicator of disease control was only evaluated in Study A, and further work to validate this finding is required.

It is also the case that both studies recruited patients with moderate to severe AE, and it will be important to evaluate the relevance of the proposed outcome measures in patients with milder disease. Nevertheless, these studies tested two different methods of data collection (paper versus electronic data capture), and evaluated outcomes in long-term studies; thus providing information as to the feasibility of this approach in future long-term trials.

### Generalisability

It is not yet clear whether the results seen in this study are applicable to the types of patients seen in primary care, who are likely to have much milder AE and may be less willing to use topical corticosteroids due to fear of side-effects. As the flare definition based on escalation of treatment, is specified on an individual basis at the start of the study, this outcome could, in theory, be used across all severity groups.

### Implications for research and clinical practice

This is the first study to have formally validated a measure of disease exacerbations or flares in AE. By concentrating on the number of days in flare, rather than the number of discreet flare episodes, we have not formally defined what constitutes the end of one flare episode and the start of another. This distinction may be important for studies that are assessing the duration of AE flares and the number of flare episodes over a period of time.

### Conclusion

Capturing disease flares in long-term AE studies is potentially burdensome for patients and researchers and remains a challenge. Nevertheless, our experiences from two large-scale, prospective studies would suggest that escalation of treatment and use of topical anti-inflammatory medications can be good indicators of long-term disease control.
